# Supplementary material for: Identification of photoperiod-induced specific miRNAs in the adrenal glands of Sunite sheep (Ovis aries)
Source: Front Vet Sci. 2022 Jul 22;9:888207. doi: 10.3389/fvets.2022.888207 (PMC9354845; doi:10.3389/fvets.2022.888207)
Supplement: Supplementary file 1 [file Data_Sheet_1.ZIP › Supplementary file/miRDeep2 file.zip!/miRDeep2 file/Novel_Structure/SP42A3/Novel_595.pdf]

Provisional ID	:	NC_019484.2_31909
Score total	:	11.7
Score for star read(s)	:	3.9
Score for read counts	:	4.2
Score for mfe	:	2
Score for randfold	:	1.6
Score for cons. seed	:	
Total read count	:	20
Mature read count	:	10
Loop read count	:	0
Star read count	:	10

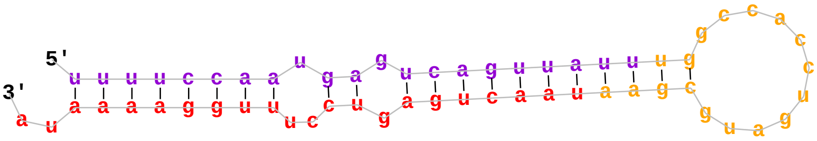

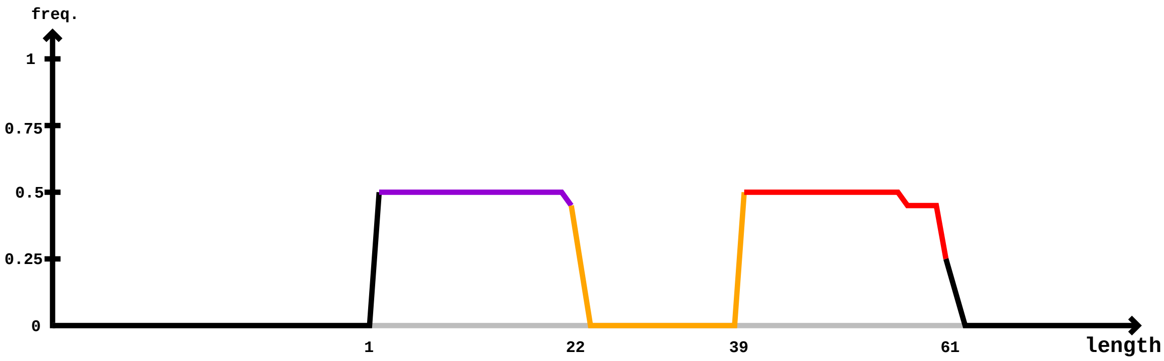

Star Mature

```
5'- ucucccuguuuuaaaacuucaacaucaggggucuuuuccaaugagucaguuauuuggccaccugaugcgaauaacugaguccuuuggaaaauacccugaugcugggaaagac   -3'   obs
    ucucccuguuuuaaaacuucaacaucaggggucuuuuccaaugagucaguuauuuggccaccugaugcgaauaacugaguccuuuggaaaauacccugaugcugggaaagac         exp
    ..((((.(((....))).....(((((((((.((((((((.((.(((((((((((..........))))))))))).))..)))))))).)))))))))..)))).....    reads     mm       sample
    ................................uuuuccaaugagucaguuau...........................................................    1         0        seq
    ................................uuuuAcaaugagucaguuauu..........................................................    1         1        seq
    ................................uuuuccaaugagucaguuauC..........................................................    3         1        seq
    ................................uuuuccaaugagucagCuauu..........................................................    1         1        seq
    ................................uuuuAcaaugagucaguuauuu.........................................................    1         1        seq
    ................................uuuuccaaugagucaguuauuA.........................................................    1         1        seq
    ................................uuuuccaaugagucaguuauuu.........................................................    1         0        seq
    ................................uuuuccaaugGgucaguuauuu.........................................................    1         1        seq
    ......................................................................uCacugaguccuuugga........................    1         1        seq
    ......................................................................uaacugaguccuuuggaaaau....................    4         0        seq
    ......................................................................uaacugaguccuuuggaaaaua...................    5         0        seq
```
